# On a New and Advantageous Mode of Preparing Alluminium

**Published:** 1856-04

**Authors:** H. Rose, J. Tyssowski

**Affiliations:** U. J. Dr.


### SELECTED ARTICLES.

### ARTICLE XIII.

### *On a New and Advantageous Mode of Preparing Alluminium.* By PROF. H. ROSE. Translated and read before the National Institute, Washington, D. C., Oct. 5, 1855. By J. TYSSOWSKI, U. J. Dr.*

SINCE the discovery of aluminium by Wöhler, Déville has recently taught us a method of obtaining it in larger connected masses, in which this metal shows qualities, which had not been observed in the metal as obtained in the form of a powder by the process of Wöhler. While in the latter form it burns with great brightness to a white clay, it may be, if fused into larger balls, heated to redness, without perceptibly oxydizing. These differences may be attributed to finer division and greater density, although according to Déville, Wöhler's metal contains some platinum which accounts for its less fusibility.

After the publication of Déville's investigations I tried also to obtain aluminium from chlorid of aluminium and sodium, by means of an additional dose of sodium. I did not follow entirely the method of Déville, but stratified the salt with the sodium and then applied heat. I however obtained no satisfactory results. Rammelsberg also followed closely the method of Déville but obtained no good results; moreover he was seldom able to prevent the bursting of the glass-tubes with which he experimented in consequence of the action of the vapors of sodium upon the chlorid of aluminium. It appeared to me that considerable time, labor, expense and long experience would be necessary in order to obtain even small quantities of this remarkable metal.

*Poggendorff's Annalen, vol. xcvi, page 152.

The application of chlorid of aluminium and its compounds with the alkaline chlorids, is particularly objectionable for the reason that they are volatile, attract moisture very readily, and therefore the access of air must be prevented when treating them with sodium.

I, therefore, early thought of substituting for the chlorid of aluminium the fluorid of aluminium, or rather the compounds of the latter with alkaline fluorids and which are known to us from the investigations of Berzelius. Berzelius pointed out the strong affinity of fluorid of aluminium for fluorid of potassium and fluorid of sodium, and that the cryolite occurring in nature is a pure combination of the fluorid of aluminium with the fluorid of sodium.

This compound, in consequence of its constituents, is as well adapted to the preparation of aluminium by means of sodium, as the chlorid of aluminium and its compound with chlorid of sodium. And in addition to this, the cryolite not being volatile, easily reducible to the finest powder, free from water, and not attracting moisture from the atmosphere, it presents peculiar advantages in comparison with the forementioned compounds.

I succeeded in fact in obtaining aluminium by heating powdered cryolite with sodium to redness in a small iron crucible much more easily than by a similar treatment of chlorid of aluminium and its compound with chlorid of sodium. The scarcity of the mineral, however, prevented me from continuing my experiments.

A short time since, I returned to them, on having obtained through Mr. Krantz, in Bonn, a considerable quantity of the purest crystals and at a trifling expense, (1 kilogram for 2 Prussian dollars.) But my zeal was exalted by the unexpected news reaching me that cryolite may be obtained here in Berlin and at incredibly low prices.

Mr. Krantz had commuuicated to me his having heard that cryolite exists in masses in commerce, yet he could not learn where. A short time since Mr. Rüdel the superintendent of the chemical manufactory of Mr. Kunheim near the Halle gate, presented to me a specimen of a white coarse powder, large quantities of which it is said have been sent from Greenland, through Copenhagen to Stattin under the name of mineral soda at three Prussian dollars per cwt. Samples of 40 lbs. had been handed to the soap-manufacturers of this place: and in fact by treating them with burnt lime, a soda-ley was prepared from it, which probably from its very admixture with alumina proved very superior for the manufacture of certain soaps.

I recognised in this powder the mineral *cryolite* of the same purity as the crystals obtained through Mr. Krantz. It dissolved perfectly (in a platinum vessel) and without leaving any insoluble matter in hydrochloric acid; the solution evaporated with sulphuric acid to dryness, showed a deposit, which heated to the expulsion of the free sulphuric acid, proved perfectly soluble in water with the aid of some hydrochloric acid. In the solution, a considerable precipitate of alumina was obtained by ammonia. A filtered solution thereof gave, after evaporation and heating, a deposit of sulphate of soda, which contained no potassium. Besides this, the powder showed the known reactions of fluorine in a very high degree.

This powder is, therefore, cryolite of great purity. Still the coarse powder which I obtained at first, was not the original form in which it comes in commerce. The cryolite arrives here in Berlin in large masses. For the purpose of preparing aluminium it must be reduced, however, to a very fine powder.

In my experiments for the preparation of the metal which I made conjointly with Mr. Weber and with important aid from him, I have used, thus far, for the most part, small iron crucibles $1\frac{3}{4}$ inches high, and $1\frac{5}{8}$ inches diameter, which I had cast in a foundery of this place. In these I stratified the fine cryolite powder with thin layers of sodium, stamped the whole pretty strongly, and covered it with a good cover of chlorid of potassium, and the crucible with a well fitting porcelain cover. Of all the fluxes, I found the chlorid of potassium the most advantageous; it has the least specific gravity, which in view of the very small density of the aluminium is of great consequence. Besides it fuses with the fluorid of sodium to a mass more fusible than the latter alone. I usually employed equal proportions of chlorid of potassium and of cryolite, and for five parts of the latter I took two parts of sodium. Ten grams of cryolite powder answered the best for the crucible.

The whole was then exposed to a powerful red heat, by means of a blowing apparatus, in which atmospheric air and illuminating gas is forced through pipes constructed after the principle of Daniell's pipe in the explosive gas-blowpipe apparatus. It appeared to be the best to continue the red heat during half an hour and no longer, keeping all the time the crucible carefully closed. Meanwhile the whole is well fused. After cooling, the contents of the crucible are best cleared by means of a chisel, which operation may be facilitated by gentle blows with a hammer on the outside of the crucible. The crucible may be used repeatedly for new operations; though finally it will fall to pieces, in consequence of the blows applied.

The fused mass is treated with water. Usually no gases or only inconsiderable and hardly perceptible quantities of gas are evolved. The small amount of hydrogen given out has the same unpleasant odor as the gas forming during the solution of iron in hydrochloric acid. The carbon comes from the minute portion of naphtha which adheres to the sodium even after drying.

In consequence of the difficult solubility of the fluorid of sodium the fused mass softens but slowly. The addition of chlorid of potassium somewhat increases the solubility. After twelve hours the lumps are softened so much that after pouring away the liquid they may be flattened with a pestle in a porcelain mortar. Larger globules of aluminium are then found, weighing from 1·3 to 0·4 grams, which are separated. I have recently found them of a weight of 0·5 grams. The smaller globules cannot be separated by lixiviation from the alumina simultaneously formed and the cryolite underlying the latter, these being heavier than the aluminium. The whole is treated cold with dilute muriatic acid. This, although it does not separate the calcined alumina, yet gives the aluminium globules their metallic lustre. They are dried, and then the alumina particles and the non-decomposed cryolite powder is separated by rubbing upon silk muslin, the little metallic globules remaining upon the tissue.

The pouring of water over the fused mass is done in platinum or silver cups. Porcelain vessels ought to be avoided, their glazing being attacked strongly by the solution of the fluorid of sodium. The solutions clarified by being left standing in a large silver cup may be evaporated in platinum cups, in order to obtain the fluorid of sodium, which, however, is mixed with a considerable portion of chlorid of potassium.

The small globules of aluminium can be fused together in a small covered porcelain crucible under a cover of chlorid of potassium by means of a blowpipe. Attempts to unite them without the use of the crucible never succeed. The small globules cannot be fused like small globules of silver, because the aluminium, although apparently it does not oxydize while heated with access of air, still it is during that process covered with an almost imperceptible pellicle of oxyd, which prevents the fusion.

The fusion under chlorid of potassium is always attended with a loss of aluminium. A globule of aluminium of 3·85 gr. weight lost by fusion under chlorid of potassium 0·05 gr. The chlorid of potassium after being dissolved in water, did not show any alumina, a small quantity of which, however, separated undissolved. Another part of aluminium unquestionably decomposed the chlorid of potassium ; chlorid of aluminium and of potassium must have volatilized by the fusion. Other metals comport themselves similarly, as copper and even silver.

I followed, therefore, the direction of Déville, and fused the globules of aluminium in a covered porcelain crucible under a cover of chloride of aluminium and sodium. The salt was first fused and then the globules were introduced into the molten salt. In this way the loss of metal is none or very minute, at the most only a few miligrams.

If the aluminium is melted under a cover of chlorid of potassium, its surface is not thoroughly even, but shows minute excavations, which is not the case if it is melted under chlorid of aluminium and sodium.

The quantities of chlorid of aluminium and sodium to be employed for this purpose, are most easily prepared by bringing the mixture of alumina and charcoal in a glass tube of as large a diameter as possible, and by introducing in it another glass tube open at both ends of a smaller diameter filled with pulverized table-salt. By heating the space containing the mixture of alumina and charcoal very strongly, and that which contains the chlorid of sodium less, while passing the chlorine gas through the tube, the vapors of the chlorid of aluminium will be absorbed so eagerly by the chlorid of sodium, that no chlorid of aluminium or only a trace of it deposits on the other parts of the apparatus. If the smaller glass tube with the chlorid of sodium has been weighed beforehand, the quantity of the chlorid of aluminium it has absorbed can be easily determined. The latter, however, is not combined equally with the chlorid of sodium; that part which was next to the mixture of clay and charcoal, contains the most of it.

I have varied the process for the reduction of aluminium in many ways, but have returned to that described. Often the sodium was placed alone on the bottom of the crucible, over it the cryolite powder, and then the chloride of potassium. In this case, copious vapors of sodium escaped, burning with a strong yellow flame, which did not take place if the sodium was cut in thin slices and stratified with the cryolite powder when the process goes forward very quietly. From the start, the incandescent crucible suddenly glows up vehemently while the decomposition of the compounds takes place. At this point the heat must not be reduced, but kept on, not however beyond half an hour. Through a longer glowing heat, a great loss would be experienced in consequence of the action of the chlorid of potassium upon the aluminium. Neither will the globules of aluminium become larger by a longer glowing heat, which was tried, up to two hours; this effect was produced only through the most intense heat possible. If, however, after the highest incandescence of the crucible, that is, after five or ten minutes, the heating ceases, the gain in aluminium is remarkably small, the metal then not having yet formed in globules, remaining in the form of a powder and burning up during the cooling of the crucible.

No greater gain is obtained if the cryolite powder be first mixed with a part of the pulverized chlorid of potassium and then stratified with the sliced sodium.

I also tried covering the cryolite stratified with sodium, with a layer of chlorid of aluminium and sodium, and heated the whole as usual. But I found no gain in this way.

I often employed, in place of chlorid of potassium, chlorid of sodium (common table-salt heated) without observing any considerable difference in the result. Only the heat has to be raised higher than when using chlorid of potassium.

The operation may also be performed in hard, fusible unglazed stone-ware crucibles of the same size as the cast iron crucibles aforesaid. At a very high temperature they only resist with more difficulty the action of the fluorid of sodium and melt in one or more places. The iron crucibles melt also, if when filled with the mixture for the preparation of aluminium they are exposed to a very intense charcoal fire.

The amount of aluminium obtained has, up to this time, been very variable, even with the same process and the same proportions of materials. The quantity of metal contained in the cryolite treated has never been reached. The latter contains 13 per cent. of aluminium. By employing 10 grms. of cryolite, a quantity which was the standard in all experiments with the small crucibles, the most favorable product was 0·8 grm. of aluminium. If, however, only 0·6 or even 0·4 grm. were obtained out of the 1·3 grm. which, according to calculation were contained in the cryolite treated, the result would still be called satisfactory. Often only 0·3 grm. and less were obtained.

These so different results depend on various circumstances, principally, however, upon the degree of heating. The greater the heat the more the small globules unite into larger ones, and the less aluminium remains in the state of powder, which could during the subsequent cooling oxydize into alumina. I succeeded sometimes in uniting by fusing in a stone-ware crucible and with a very great heat, almost the whole quantity of the aluminium obtained into one single globule of 0·5 grm. weight.

The heat, which I am enabled to give by means of the blowpipe apparatus, is not equal at all times of the day, depending as it does upon the varying pressure to which the illuminating gas is subject in the gas metres of the city. But that a great loss of aluminium is caused through a very slow cooling under access of air which favors the oxydation of the small particles of the reduced metals, is proved by the following experiment.

In a large iron crucible 35 grams of cryolite powder were stratified with 14 grams of sliced sodium, and the whole covered with a thick stratum of chlorid of potassium. The crucible covered with a porcelain cap, was placed in a larger earthen-ware crucible, also covered, and exposed during an hour to a good charcoal fire in a well drawing wind furnace, and cooled as slowly as possible. The product in aluminium was in this case remarkably small, only 0·135 gram. in globules being separable from the molten mass.

The varying result originates also in this, that through the varying stratification of the sodium with the cryolite powder, a large amount of the latter often escapes decomposition. The more sodium employed, the less is this the case; but because of the great difference in the price, I never took more than 4 grm. of sodium for 10 grms. of cryolite.

To avoid the loss resulting from the oxydation of the pulverulent aluminium during cooling, I tried some other methods of operation. 20 grms. of cryolite were strongly heated in a gun-barrel exposed to a current of hydrogen, and then the vapors of 8 grms. of sodium passed over it. This was accomplished simply by introducing the sodium placed on a sheet-iron pan in that part of the gun-barrel which stood out of the furnace, and pushing the same in the fire only at the moment when the cryolite powder reached its highest point of incandescence. The operation went on admirably. I permitted the whole to cool in the hydrogen current. After lixiviation in water, in which the fluorid of sodium dissolved with great difficulty, I obtained a great quantity of a black powder, which for the most part consisted of iron, and which in its solution in hydrochloric acid showed but little alumina.

The smallness of the products which I obtained in most instances, must not deter from further investigations. They are the results of the first experiments in which I have been engaged and for a short time. Now when the cryolite may be obtained at so low a price, and as sodium (for the increased facilities of reducing which we are so much indebted to Déville) will also in future be much cheaper, any one may undertake to produce aluminium, and certainly in no distant time methods will be found that will afford satisfactory results.

Anyhow, I am of the opinion that the cryolite among all the compounds of aluminium will prove the most eligible for the production of aluminium. It possesses so many advantages over the chlorid of aluminium and the chlorid of aluminium and sodium, that it would be employed with the greatest benefit even if its price were considerably higher.

Aluminium has yet hardly been obtained directly from alumina. Potassium and sodium appear to effect the reduction of the metallic oxyds only when the nascent potassium or sodium is at hand to combine with a part of the oxyd not yet reduced. Pure potash and soda, the properties of which are almost unknown to us, do not appear to form at that moment. And as alumina can very readily combine with alkalies to form an aluminate, it should be inferred, that the reduction of alumina through the alkaline metals might succeed in the end.

But even if it should become possible to obtain aluminium directly from alumina, still cryolite may for a long time be employed for the purpose, unless its price should rise immoderately. Nature furnishes this substance in a state of rare purity, the aluminium is combined in it only with fluorine and sodium—two substances which cannot act injuriously during the production of the metal. Clay or aluminous earth is however seldom found in a pure state, and always of great density. To reduce aluminous earth in large quantities from its compounds and to purify it from substances which could act injuriously during the production of aluminium—would be attended with great difficulties.

The globules of aluminium obtained by me are for the most part so extensible, that they may be flattened very considerably and rolled into the thinnest sheets without their showing fissures on the sides. They have at the same time a strong metallic lustre. On the contrary some few masses, found on the bottom of the crucible and sometimes adhering to it and which had no globular form, showed fissures when rolled, and differed somewhat in color as well as in lustre. They evidently were not as pure as the great majority of the globules, and probably contain some iron.

A large globule of aluminium of 3·8 grm. weight, having been sawn in two, it could be plainly seen that the metal was brittle for about half a line from the outside; but inside it was soft and pliable. Sometimes excavations are found in the interior of the globules.

According to the observations of Déville I happened also to obtain the aluminium in crystalline form. One of the greater globules became in cooling radiated with crystals on its under surface. Déville believes he has obtained regular octahedral crystals, but does not, however, assert this positively. According to the investigations of my brother, the crystalline structure does not appear to belong to the regular system.

Trying to melt a larger globule, accidentally contaminated, after being rolled without flux, before the heat rose so as to melt the whole, small globules went floating on the surface. The impure aluminium is less fusible than the pure which is mixed with it, expands in melting and rises out of the mass not yet fused. It is a phenomenon similar to that observed by Mr. Schneider with impure bismuth.

I have stated that the cryolite has been employed here in Berlin under the name of mineral soda for the preparation of caustic soda-ley, which owing to its aluminous earth appears eminently adapted to the manufacture of soap. In fact the pulverized cryolite is decomposed entirely if boiled in this condition with caustic lime and water. The fluorid of calcium thus generated contains no aluminous earth, this being entirely dissolved in the hydrate of soda, which again is free from fluorine or shows but a trace of it.

*Aluminium and Silicium.*—The memoir of M. H. Rose on the preparation of aluminium from cryolite has been the means of important improvements in this manufacture. Déville had recognised that with the addition of fluorid of calcium to the bath of the double chlorid of aluminium and sodium, aluminium may be obtained, while it is not possible with the chlorid alone. The fluorids are, therefore, excellent solvents.

A mixture of alumina and fluorid of sodium wet with fluohydric acid may be decomposed by sodium, and aluminium obtained. A mixture of fluorid of potassium and fluorid of sodium is an excellent solvent. It is very fusible and is capable of dissolving much silica, some titanic acid, and a little alumina. This addition of foreign matter even augments the fusibility and renders the fusion as liquid as water. By the aid of the galvanic pile, silicium may be obtained, which forms an alloy with the electrodes unless they are of platinum.

M. Déville has satisfied himself that alumina is not decomposed by sodium, while silica is decomposed. He has even prepared sodium by bringing together capillary glass and sodium in vapor. But the great difficulty in these experiments is in the nature of the vessels used for the experiments and the alterability of the electrodes. For gas carbon is dissolved rapidly in the baths of fluorids when it is used in the preparation of silicium.

Aluminium is manufactured now on quite a large scale at Amfréville near Rouen. The vapors set free in this process are very noxious, as they consist of chlorid of silicium, chlorid of aluminium, chlorid of sulphur and chlorohydric acid. These are disposed of by interposing in their passage a furnace of lime, heated by an adjoining fire, into which, through the draught of the chimney, come the vapors of the reducing process, and also the flame that heats the limestone.—*Am. Jour. of Med. Sci.*

